# Food allergy education: teen learning preferences

**DOI:** 10.1186/1710-1492-10-S2-A65

**Published:** 2014-12-18

**Authors:** Claire R Unruh, Cathy A Gillespie, Nancy L Ross, Allan B Becker

**Affiliations:** 1 Department of Pediatrics and Child Health, University of Manitoba, Winnipeg, Manitoba, R3A 1S1, Canada; 2 Children’s Allergy and Asthma Education Centre, Winnipeg, Manitoba, R3E 0Z2, Canada

## Background

Food allergic teens are at increased risk for fatal anaphylaxis[1]. Food allergy education is needed to address the transition of care from their parents to the teens. Teen input into education approaches is essential in order to effectively develop programs that will modify behavior. Allergy educators will need to be familiar with effective approaches to education for this important population.

## Methods

Teens with food allergy were invited into focus groups in our education centre as a preliminary step to determine their preferred learning styles to begin development of effective educational resources for teens. Semi-structured interviews were conducted, digitally recorded, transcribed and reviewed for themes.

## Results

16 teens (mean 16 yr.) participated in three focus groups facilitated by a food allergy educator. Common themes from these interviews highlighted the need for different methods of communication (both from and to the teens) and behavioral approaches to self-advocacy, risk assessment and reduction, reaction recognition and treatment. In-depth information about allergic reactions and on-going research were also of interest. Learning preferences included spatial, auditory, verbal and kinesthetic style examples. All groups emphasized a need for some hands-on classroom experiences, including: practice with auto-injectors, playing out different scenarios, and distance and mobile information.

The teens expressed interest in small group participation where they could voice their opinions, have questions answered, and comfortably communicate with others. Many teens said they liked hybrids of different learning styles, such as auditory and visual instruction followed by hands-on experience in the classroom. Most teens preferred a group facilitator expert in food allergies and/or who had food allergies, educator skills, and could relate to younger people. Online and mobile learning was of interest but most had not yet used these resources.

## Conclusion

Teens are interested in small group interactive education with hands-on experience, as well as mobile-based learning. Food allergy topics must be adapted to teen specific situations, and a teen program needs to include a variety of approaches to connect with teens. These focus groups have led to the deployment of an online survey for teens to acquire a greater breadth of feedback for topics and teens’ learning preferences.
